# Latent Multidimensional Organization of White Matter Microstructural Aging: A Generative Adversarial Network Disentanglement Study in a Clinically Defined Reference Cohort

**DOI:** 10.3390/brainsci16101076

**Published:** 2026-10-07

**Authors:** Yanxue Li, Hongjian Gao, Lan Lin, Min Xiong

**Affiliations:** 1 Department of Biomedical Engineering, College of Chemistry and Life Science, Beijing University of Technology, Beijing 100124, China; liyanxue@emails.bjut.edu.cn (Y.L.); gaohongjian@bjut.edu.cn (H.G.); 2 School of Science, North China University of Technology, Beijing 100144, China

**Keywords:** brain aging, deep learning, diffusion MRI, heterogeneity, influencing factors, UK Biobank

## Abstract

**Highlights:**

**What are the main findings?**

- Three reproducible white matter aging patterns were identified with distinct microstructural features.
- The patterns showed partially shared and partially independent associations with cognition, lifestyle, systemic health, and disease.

**What are the implications of the main findings?**

- White matter aging is a multidimensional rather than uniform process.
- Disentangling aging patterns may improve characterization of individual differences in brain aging.

**Abstract:**

**Background:** White matter (WM) aging is commonly characterized as a largely unitary process of progressive microstructural decline, yet substantial interindividual variation suggests that this view may obscure distinct biological dimensions. Whether WM aging retains a multidimensional organization after minimizing variation attributable to overt clinical pathology remains unknown. **Methods:** We constructed a clinically defined aging reference cohort (CDAR; *n* = 17,310) from the UK Biobank (UKB) by excluding overt clinical pathology and analyzed 144 skeleton-based WM microstructural metrics. An additional 10,272 participants with overt clinical diagnoses were included for downstream association analyses, yielding a total analytic sample of 27,582. Surreal-GAN was applied to disentangle latent dimensions of WM aging, with reproducibility-based model selection and cross-sample and cross-sex validation. The biological relevance of the identified dimensions was evaluated through associations with cognitive, lifestyle, peripheral, and disease-related outcomes. **Results:** Three reproducible and coexisting dimensions of WM aging emerged, comprising distinct spatial configurations rather than a single diffuse aging axis. R_1_ displayed an anterior-vulnerable/posterior-preserved gradient, R_2_ reflected a widespread diffuse aging load, and R_3_ showed a dissociation between the limbic–frontal system and sensorimotor pathways. The three patterns exhibited good cross-sample (r = 0.887–0.935) and cross-sex (r = 0.891–0.937) reproducibility, with strong bilateral hemispheric correspondence (r > 0.86). All three patterns were associated with age but exhibited partially shared and partially independent associations with cognition, lifestyle factors, peripheral phenotypes, and disease risk. **Conclusions:** WM aging is not adequately captured by a single neuroanatomical process. Even after minimizing overt clinical pathology, it retains a reproducible multidimensional organization, with partially shared and partially independent phenotypic profiles across aging dimensions. These findings suggest that individual differences in WM aging reflect partially separable biological processes rather than variation along a single aging axis.

## 1. Introduction

During aging, the brain undergoes a series of functional and structural changes [1], and these changes exhibit marked interindividual heterogeneity [2]. Rather than following a uniform trajectory, brain aging is shaped by the combined influence of multiple biological processes operating at different spatial and temporal scales. This heterogeneity is jointly influenced by a range of pathological factors (e.g., neurodegenerative diseases such as Alzheimer’s disease and Parkinson’s disease) [3,4] and non-pathological factors (e.g., lifestyle, genetic background, and environmental exposures) [5,6,7,8]. The interaction of these factors not only leads to substantial variability in the rate and spatial distribution of brain aging across individuals, but may also be reflected in the coexistence of multiple neuroanatomical patterns within the same individual. Moreover, alterations in specific brain regions or spatial patterns during brain aging partly overlap with prodromal brain changes observed in certain neurodegenerative diseases, making it increasingly difficult to distinguish physiological aging from covert pathological alterations. Emerging evidence further indicates that even among healthy individuals, distinct aging phenotypes exist, such as the coexistence of accelerated brain aging (ABA) and resilient brain aging (RBA) [9], suggesting that such macroscopic individual diversity stems from the distinct, individualized mixtures of underlying neuroanatomical patterns. Despite growing recognition of this complexity, existing analytical frameworks primarily characterize population-average trajectories or assign individuals to mutually exclusive subtypes, failing to represent the multidimensional and coexisting nature of brain aging at the individual level. This highlights an imperative need for interpretable analytical approaches capable of disentangling these interwoven neuroanatomical variations within each individual.

Currently, an increasing number of researchers are integrating neuroimaging with artificial intelligence to investigate the heterogeneity of brain aging, introducing new analytical perspectives to the field [10]. Gray matter (GM) morphometric analysis based on high-resolution T1-weighted images has long served as the primary imaging framework for characterizing macrostructural brain aging. Early studies primarily relied on pattern classification or clustering approaches to identify distinct brain aging phenotypes, such as [11] ABA or RBA, or to further subdivide individuals with similar aging characteristics into discrete subgroups [12]. While these approaches have provided valuable insights into population heterogeneity, they generally assign each individual to a single category and therefore have limited ability to represent the potentially multidimensional nature of brain aging variability. Recent advances in representation learning have provided an alternative analytical framework. Rather than partitioning individuals into mutually exclusive subgroups, multidimensional disentanglement approaches based on generative adversarial networks (GANs) represent each individual as a continuous combination of multiple latent dimensions, thereby offering a more flexible characterization of heterogeneous neuroanatomical variation. These approaches have been successfully applied to identify disease-related brain atrophy patterns [13] and have recently been extended to brain aging [14]. Yang et al. [15] leveraged an improved Surreal-GAN model in a large-scale multicenter cohort to identify five dissociable but coexisting brain aging atrophy patterns from macrostructural volume features of whole-brain GM and white matter (WM), and systematically elucidated the associations of these patterns with genetics, cognition, lifestyle, and multiple chronic diseases. Notably, although the input features in that study included both GM and WM volumes, the five learned dimensions all manifested as atrophy patterns in GM regions, while WM injury only appeared as WM hyperintensity (WMH) volume in the form of an external validation indicator. This study represents an important advance by demonstrating that brain aging heterogeneity can be modeled as multiple latent neuroanatomical dimensions rather than discrete subtypes.

Compared with the rapid progress in research on GM heterogeneity, the investigation of WM microstructural heterogeneity remains in its infancy. Existing diffusion MRI studies have systematically characterized age-related WM alterations at multiple spatial scales, including the voxel level [16] and the tract level [17], and have consistently demonstrated associations of decreased fractional anisotropy (FA), increased mean diffusivity (MD), and other metrics with aging, cognitive decline, and cerebrovascular risk [18]. However, describing the average trajectory of WM aging does not necessarily capture the biological complexity of the WM system.

WM is a complex system composed of multiple fiber pathways with distinct functional and anatomical properties. Major WM tracts, including the frontal association fibers, corpus callosum, cingulum, and superior longitudinal fasciculus, differ substantially in their developmental origin, myelination profile, vascular supply, and functional specialization [19]. Such biological diversity suggests that different fiber systems may exhibit distinct patterns of age-related vulnerability rather than undergoing uniform degeneration. Evidence supporting this regional vulnerability has begun to emerge. Cross-sectional and longitudinal MRI studies have shown that the rate of FA decline is generally greater in prefrontal association fibers than in primary motor and sensory pathways [20], while the anterior corpus callosum appears more susceptible to aging than its posterior counterpart [21]. These observations indicate that WM aging is spatially heterogeneous, with different fiber systems exhibiting distinct aging characteristics.

Crucially, these regionally heterogeneous patterns also imply that the distinct degeneration rates of various fiber systems could combine uniquely within each person, resulting in highly individualized combinations of microstructural degradation. Nevertheless, existing studies have almost exclusively characterized such heterogeneity at the population level through regional comparisons or tract-specific statistical analyses. Whether these regionally heterogeneous alterations can be represented as multiple latent neuroanatomical patterns at the individual level remains largely unknown. Furthermore, the multidimensional decomposition framework that has demonstrated considerable success in modeling GM macrostructural heterogeneity has not yet been extended to diffusion MRI-derived WM microstructural features. Consequently, the latent organization of WM microstructural aging remains poorly understood.

Characterizing heterogeneous patterns of WM aging requires addressing another fundamental challenge beyond representation learning: the difficulty of separating physiological aging from covert pathological alterations. A longstanding perspective in brain aging research, often summarized as the Health-to-Disease Continuum (HDC), considers normal aging and neurodegenerative processes as different stages of a shared biological process [22]. While this framework captures the close relationship between aging and disease, it also highlights an important methodological challenge: in heterogeneous populations, age-related neuroanatomical variation may be confounded by subclinical pathological processes that share overlapping imaging manifestations. This challenge is particularly relevant for WM, where microstructural alterations are highly sensitive to vascular burden, metabolic disorders, and other systemic pathological conditions. As a result, pathological and physiological WM alterations frequently co-occur spatially, making it difficult to attribute the observed imaging patterns specifically to normal aging. Therefore, reducing pathology-related variation represents an important prerequisite for investigating the intrinsic heterogeneity of WM aging. To address this issue, based on the Dissociable Aging-Pathology Hypothesis (DAPH) [23,24], rather than introducing a new framework, we adopted a clinically defined aging reference (CDAR) strategy, which minimizes overt pathology-related variation through stringent clinical screening. Building upon this low-confound aging reference, we applied the validated Surreal-GAN framework to diffusion MRI-derived WM microstructural features. We aimed to determine (i) whether, after minimizing overt clinical pathology, WM aging remains decomposable into multiple reproducible latent dimensions, and (ii) whether these dimensions show differential associations with cognition, lifestyle, peripheral phenotypes, and disease risk. We also asked whether these dimensions provide information beyond chronological age.

## 2. Materials and Methods

### 2.1. Data and Preprocessing

#### 2.1.1. Study Population and Imaging Derived Phenotypes (IDPs)

The data for this study were obtained from the UK Biobank (UKB) cohort, which includes over 500,000 volunteers aged 40–69 years from across the United Kingdom [25]. As part of the UKB study, its imaging extension project was launched in 2014 and has since collected neuroimaging data from a subset of participants; this dataset has grown into one of the largest and most comprehensive neuroimaging datasets to date [26]. It encompasses six imaging modalities, including T1-weighted MRI (T1w MRI), T2-weighted fluid-attenuated inversion recovery (T2-FLAIR), susceptibility-weighted imaging (SWI), diffusion MRI (dMRI), resting-state functional MRI (rfMRI), and task functional MRI (tfMRI), offering a comprehensive assessment of brain structure, microstructure, and functional activity. The UKB study received ethical approval from the North West Multi-Centre Research Ethics Committee (REC reference: 11/NW/0382), and written informed consent was obtained from all participants prior to data collection. The present analyses were conducted under the approved UKB application (number: 68382).

The dMRI data in the UKB were collected across three imaging centers: initial acquisition began in Manchester, with two additional centers (Newcastle and Reading) added in 2017. To ensure data consistency, all three sites were equipped with identical 3T Siemens Skyra scanners (Siemens Healthcare GmbH, Erlangen, Germany) running the VD13A SP4 software platform and a dedicated 32-channel receive head coil. The dMRI data were acquired using a multi-shell acquisition strategy (b = 0, 1000 and 2000 s/mm^2^; 50 diffusion-encoding directions per non-zero shell) at a spatial resolution of 2 × 2 × 2 mm, to enable comprehensive characterization of WM microstructure. In addition, the UKB team implemented a centralized processing pipeline to convert raw images into IDPs, encompassing diffusion tensor metrics, microstructural model parameters, and tractography-based connectivity measures. Detailed technical specifications can be found in the official UKB documentation (https://biobank.ctsu.ox.ac.uk/crystal/crystal/docs/brain_mri.pdf, accessed 20 September 2025).

The dMRI preprocessing followed the UKB standard image processing pipeline, which used FSL 5.0.10. Gradient nonlinearity distortion correction was applied first, followed by B_0_ field map estimation and echo planar imaging (EPI) distortion correction using the FMRIB Software Library (FSL) TOPUP tool on the reverse phase-encoded b = 0 volumes. Subsequently, eddy current distortion, head motion, and slice outliers were corrected using the FSL Eddy tool, yielding high-quality, distortion-corrected and motion-corrected diffusion-weighted images. For microstructural parameter estimation, three metrics were evaluated: fractional anisotropy (FA), mean diffusivity (MD), and isotropic water volume fraction (ISOVF). FA and MD were derived from diffusion tensor fitting on the b = 1000 shell data, computed voxel-wise using the FSL DTIFIT tool. ISOVF was obtained by fitting the Neurite Orientation Dispersion and Density Imaging (NODDI) model jointly to the dual-shell data (b = 1000 and 2000 s/mm^2^) using the AMICO (Accelerated Microstructure Imaging via Convex Optimization) algorithm [27], which substantially improved model fitting efficiency through a convex optimization strategy.

To perform group-level spatial comparisons, the Tract-Based Spatial Statistics (TBSS) method [28] was implemented. In brief, each participant’s FA map was nonlinearly registered to the standard Montreal Neurological Institute 152 (MNI152) space using the FMRIB’s Nonlinear Image Registration Tool (FNIRT). On this basis, a mean WM skeleton was constructed, representing the central portions of the major fiber tracts common to all participants. Subsequently, the deformation fields estimated from the nonlinear registration were applied to the MD and ISOVF maps, ensuring consistent spatial localization of all diffusion parameters in standard space. Finally, the mean FA skeleton was projected onto each participant’s individual FA, MD, and ISOVF maps to extract skeletonized parameters. Regional quantitative analysis was conducted using the Johns Hopkins University (JHU) WM atlas [29], which parcellates the WM skeleton into 48 standard tract regions. For each region, the mean values of FA, MD, and ISOVF on the skeleton were calculated. Accordingly, a total of 144 skeleton-based WM microstructural IDPs (3 diffusion metrics × 48 WM regions) were extracted from each participant.

#### 2.1.2. Non-Imaging Derived Phenotypes (Non-IDPs)

To complement the imaging data, we retrieved a broad range of non-IDPs from UKB, based on brain aging-related factors reported in the gerontological literature. A total of 262 candidate non-IDPs were compiled and categorized into seven domains according to their substantive characteristics: cognition (26), blood assays (61), physical measures (29), lifestyle and environment (86), psychosocial factors (16), sociodemographic characteristics (34), and early-life factors (10). Each variable was categorized as continuous, count, binary, or ordinal based on its original UKB data coding and empirical distribution. During data preprocessing, special categorical codes indicating non-substantive responses (e.g., “prefer not to answer”, “do not know”) were uniformly recoded as missing values. The direction of scales was harmonized to ensure that all quantitative scores were directionally consistent and had a clear biological interpretation. To maximize sample size and preserve statistical power, subsequent association analyses adopted a model-specific complete-case strategy (also referred to as an available-case approach across different models). Specifically, each statistical model was fitted using the maximal subset of participants with complete observations for all covariates and variables required for that specific model. This approach accounts for the varying missingness patterns inherent across different UKB sub-questionnaires and laboratory assays. To ensure transparency of results, the actual effective sample size is explicitly reported alongside the effect estimates for each regression model.

### 2.2. Discovery of Neuroanatomical Patterns via Surreal-GAN

#### 2.2.1. Model

We adopted Surreal-GAN [15] to characterize the spatially distributed and temporally evolving patterns of WM microstructural aging. Unlike traditional regression models, Surreal-GAN learns a multimodal mapping from a reference group (REF) to a target group (TAR) through an adversarial game between a generator and a discriminator, while introducing an inverse mapping function (g) to reconstruct latent variables from the generated data. This constrains the model to capture discriminative and interpretable brain imaging features, thereby effectively decomposing the intrinsic neuroanatomical heterogeneity of the aging brain into multiple latent dimensions (R-indices), which quantify the expression levels of different structural change patterns.

#### 2.2.2. Study Design and Cohort Definition

To establish discrete age anchor distributions, we employed a discretized age grouping strategy. The REF group was defined as cognitively and pathologically clear participants aged ≤ 55 years, representing the pre-aging brain state, whereas the TAR group comprised individuals aged ≥ 65 years, representing the late brain aging state. Individuals aged 55–65 years were excluded from both the REF and TAR groups. The selection of these thresholds was based on three considerations: (i) midlife to late life (55–65 years) marks a transitional period of accelerated brain structural change [30]; (ii) Surreal-GAN requires sufficient distributional contrast between groups to identify robust structural patterns; and (iii) more extreme cutoffs in UKB would compress the sample size and affect training stability.

The CDAR cohort was constructed by rigorously excluding overt clinical pathology, thereby providing a data foundation for the model to learn WM aging patterns with reduced pathological confounding. Exclusion from the CDAR cohort was based on the following four principles: (1) diseases that directly affect brain structure; (2) known cerebrovascular-related diseases; (3) chronic systemic diseases known to cause persistent neuroinflammation; and (4) severe central nervous system injury. Participants were excluded if, at any time point on or before the date of imaging, their International Classification of Diseases, 10th Revision (ICD-10) records contained a disease diagnosis meeting any of the above four exclusion principles (for detailed exclusion criteria, see Supplementary Table S1). After screening, participants without any of the above exclusion diagnoses constituted the CDAR cohort; others were classified as non-CDAR individuals. Unlike a true healthy control group, which would require the absence of all subclinical and preclinical pathology, the CDAR cohort was defined operationally to minimize major pathological confounders. Therefore, CDAR does not rule out covert subclinical pathology, early protein deposition, long-term medication effects, or undiagnosed small vessel disease. The non-CDAR-REF group was excluded because it did not align with the study’s aim of modeling WM aging under minimized overt clinical pathology. The final CDAR cohort comprised 17,310 participants. In addition, 10,272 non-CDAR adults with overt clinical diagnoses were included for downstream disease association analyses, yielding a total analytic sample of 27,582 participants.

**Figure 1:**
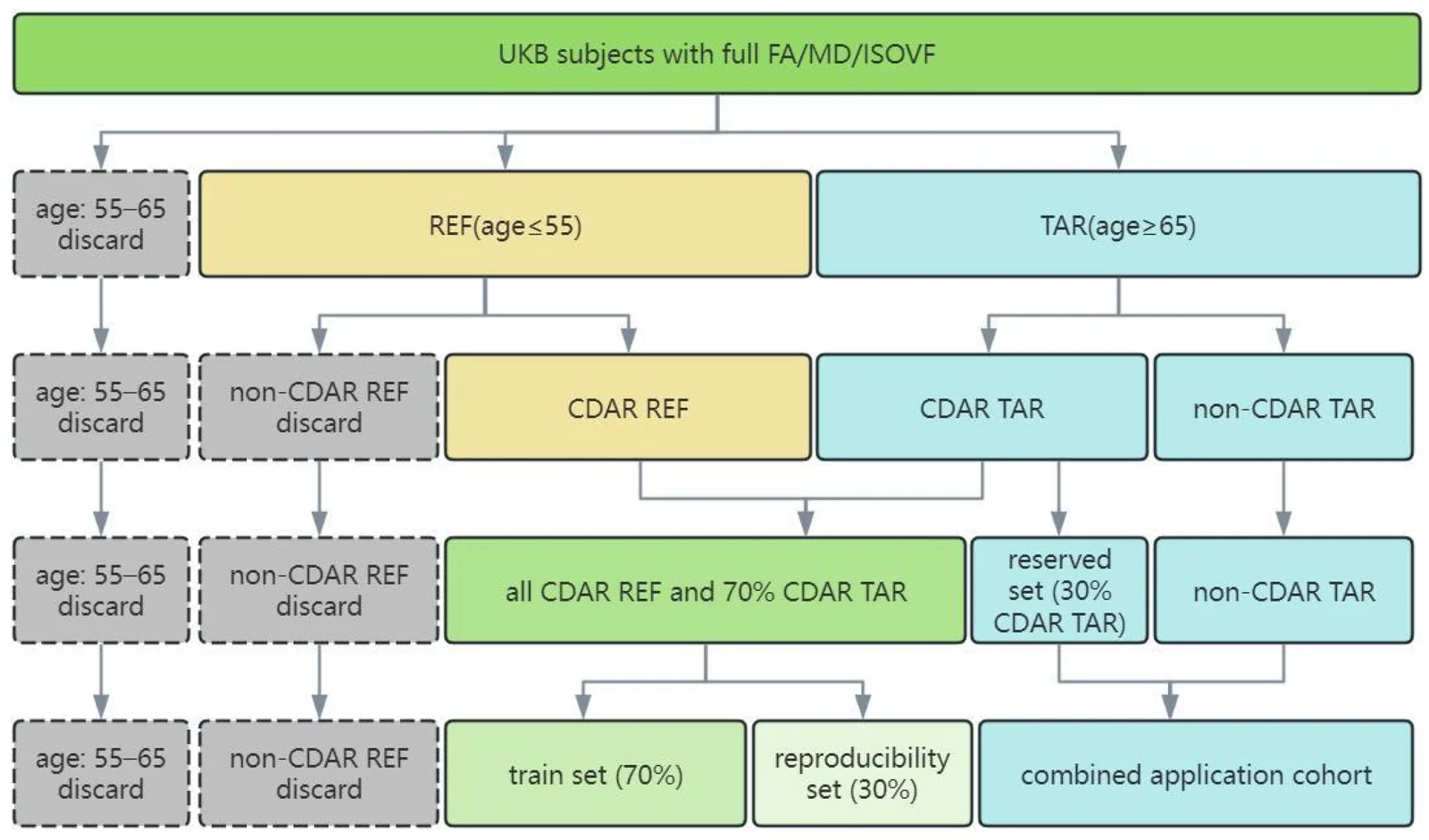
Two-stage stratification and data partitioning strategy.

Because Surreal-GAN models the contrast between two discrete age anchors rather than a continuous age spectrum, R-indices represent the projection values of individual brain features onto latent axes derived from the REF-vs-TAR comparison. To ensure unbiased evaluation and internal validation, the study population was partitioned using stratified random sampling (Figure 1): 30% of the CDAR-TAR group (*n* = 3676) was set aside as a reserved set and did not participate in the initial model construction phase; the remaining CDAR cohort (comprising the entire CDAR-REF group and the remaining 70% of the CDAR-TAR group) was further subdivided—70% of these were allocated to the training set (*n* = 9543) for training models to capture brain aging patterns, and the other 30% (*n* = 4091) served as a reproducibility set, used to independently retrain a new model to assess the reproducibility of the identified aging patterns across different samples. It should be noted that, given the weakly supervised nature of Surreal-GAN, no independent validation set was established in this study. Hyperparameter selection and determination of the optimal model were achieved by repeatedly training models separately within the training framework and maximizing inter-model consistency (R_indices_corr_); model reproducibility was employed in this process as a surrogate for conventional generalization error. After model development, the optimal model was applied to the combined application cohort (*n* = 13,948)—formed by merging the reserved set (*n* = 3676) with the non-CDAR-TAR participants (*n* = 10,272)—to elucidate the neuroanatomical characteristics of the aging patterns and to conduct downstream association analyses with various phenotypic variables and disease risks. It should be noted that no independent external healthy control group was used; the CDAR cohort itself served this role, with CDAR-REF and CDAR-TAR as the younger and older anchors.

#### 2.2.3. Model Training and Hyperparameter Optimization

To control for confounding factors, imaging features were first adjusted for sex using the REF group as the reference population. The resulting residuals were rescaled to achieve a mean of 1 and a standard deviation of 0.1, ensuring numerical stability during adversarial training. Ultimately, each participant was represented by a 144-dimensional microstructural feature vector as input to Surreal-GAN. The model was trained on the CDAR cohort, with the REF and TAR groups providing the baseline and target distributions, respectively. We performed a grid search over three key hyperparameters: number of latent patterns C ∈ [2, 5], orthogonality constraint λ ∈ [0.05, 1.6], and change magnitude regularization γ ∈ [0.1, 8]; the remaining hyperparameters were kept at their default values (α = 0.02, κ = 80, ζ = 80, μ = 500, η = 6). For each hyperparameter configuration, the model was trained for 30,000 epochs (batch size = 300), and checkpoints were saved every 1500 epochs when both the monotonicity loss and the reconstruction loss fell below 0.003. To account for stochastic optimization, each candidate configuration was trained independently across 20 random initialization seeds. The hyperparameter combination yielding the highest mean pairwise model consistency R_indices_corr_ across runs was selected as optimal. Under this optimal setup, the model was retrained across 40 independent runs; the single model exhibiting the highest correlation with the ensemble average pattern was retained as the final model for downstream evaluation. For the analyses performed in this study, Surreal-GAN (version 0.1.1) was implemented in Python 3.11 with PyTorch 2.7.0. All final results reported in this manuscript were generated using a workstation (HP Inc., Palo Alto, CA, USA) equipped with an Intel Xeon w3-2423 CPU (2.11 GHz), 64 GB RAM, and an NVIDIA GeForce RTX 4070 Ti GPU (12 GB).

#### 2.2.4. Stability and Reproducibility Assessment

Surreal-GAN outputs multidimensional continuous variables without a gold standard, making it impossible to assess generalizability and stability with a single quantitative metric. Therefore, we implemented a multi-stage validation strategy to ensure the reproducibility of the R-indices: (1) run-to-run consistency, repeated training under different random seeds and evaluation via R_indices_corr_; (2) cross-sample reproducibility, retraining the model with the same hyperparameters on the reproducibility set, aligning the new R-indices with the original ones via optimal assignment, with high correlation confirming cross-sample robustness; (3) cross-sex reproducibility, assessed both within and across sexes. Sex-specific models were trained with the same hyperparameters and evaluated in four scenarios: male-trained model applied to male samples, female-trained model applied to female samples, male-trained model applied to female samples, and female-trained model applied to male samples. In each scenario, the resulting R-indices were correlated with those from the main model in the same samples after optimal assignment, with high correlations indicating cross-sex generalizability.

### 2.3. Statistical Analysis

The analyses were conducted at two complementary levels. First, centered on the reserved set, the WM microstructural anatomical features of each aging pattern were characterized, which provided a clinical reference benchmark less confounded by disease-related variation. Second, based on the entire combined application cohort, we examined the associations between these patterns and a broad spectrum of multidimensional phenotypes, and systematically quantified the extent to which individual brain aging patterns deviate from this reference benchmark under different disease states. All statistical analyses were performed using R 4.3.3 and IBM SPSS Statistics 27.0.1.0.

#### 2.3.1. Anatomical Characteristics of WM Microstructure

The analyses in this section were conducted in the reserved set to characterize the WM microstructural anatomical features of each aging pattern in clinically screened individuals. Before examining specific anatomical features, we first tested whether the identified aging patterns captured additional neuroanatomical variance beyond chronological age, to assess whether they provided information beyond age-related variation rather than simply reflecting age redundancy or the preference of the model for a low-dimensional solution. Nested model comparisons were conducted using a baseline model incorporating age, sex, and years of education as predictors. We evaluated whether adding the R-indices significantly improved model fit based on changes in ΔR^2^, ΔAIC, and likelihood ratio tests with Bonferroni-corrected *p*-values.

Following the nested model validation, we employed generalized linear models (GLMs) to examine the associations between each pattern and WM microstructure (144 metrics derived from DTIFIT and NODDI), with sex, age, years of education, and all other R-indices included as covariates. To assess the robustness of these associations to potential confounders, we additionally performed sensitivity analyses including imaging centre and dMRI head motion as covariates. Since Surreal-GAN allows correlations among R-indices, including them simultaneously in the same model may introduce multicollinearity; therefore, variance inflation factor (VIF) was used as a diagnostic tool, with VIF < 5 established as the threshold for acceptable multicollinearity and stable regression coefficient estimation. To control the false positive rate due to multiple comparisons, all raw *p*-values were subjected to Bonferroni correction, with a significance threshold of corrected *p* < 0.01 (corresponding to uncorrected *p* < 6.94 × 10^−5^ across 144 metrics). For each aging pattern, we report its standardized regression coefficient (β) and 95% confidence interval with respect to each WM metric.

#### 2.3.2. Analysis of Associated Factors

All analyses in this section were performed in the combined application cohort using generalized linear models (GLMs). In the primary models, each R-index was entered separately as the predictor, with age, sex, and years of education included as covariates. To determine whether the phenotypic associations were independent of the other R-indices, we further performed joint models including all three R-indices simultaneously, with additional adjustment for imaging centre and dMRI head motion. For variables of different data types, we selected appropriate regression models according to the characteristics of each outcome variable: linear regression for continuous variables; binary logistic regression for binary variables; ordinal logistic regression for ordinal variables; and for count variables, overdispersion was first assessed, followed by the selection of Poisson regression or negative binomial regression depending on the presence of overdispersion. Given the large number of variables examined, this study was considered exploratory rather than hypothesis-confirmatory. To control for multiple comparisons, all results were subjected to Bonferroni correction, with the significance threshold set at corrected *p* < 0.01 (corresponding to uncorrected *p* = 3.82 × 10^−5^). All statistical computations were performed using R 4.3.3.

#### 2.3.3. Disease Association Analysis

To investigate whether the identified aging patterns were associated with the presence of different disease categories, we defined the following seven disease categories based on the ICD-10 diagnostic codes of the non-CDAR-TAR participants in UKB: diabetes, hypertension, ischemic heart disease (IHD), chronic obstructive pulmonary disease (COPD), stroke, mental disorders, and neurodegenerative diseases. Given the limited sample size for certain diseases, Alzheimer’s disease, Parkinson’s disease, multiple sclerosis, and various cognitive disorders were merged into “neurodegenerative diseases,” while depression, bipolar disorder, schizophrenia, anxiety disorders, and obsessive–compulsive disorder were grouped as “mental disorders.” Participants meeting diagnostic criteria for multiple diseases were included simultaneously in each applicable disease group, and the reserved set served as the clinically screened healthy control. This grouping strategy represents a pragmatic choice, but it comes at the cost of disease specificity and may obscure heterogeneity within subtypes; therefore, the observed effect sizes should be interpreted as category-level associations. Additionally, since UKB participants were predominantly drawn from the general population and not recruited from clinical or institutional settings, with most conditions well controlled, the observed effect sizes may underestimate those observed in clinically severe populations. Multiple linear regression was employed, with age and sex as covariates, to estimate the association between disease status and aging patterns. The Bonferroni correction was applied to control for multiple comparisons (significance threshold *p* < 0.01). To further assess the robustness of the associations, a sensitivity analysis was performed by additionally including body mass index (BMI), smoking status, education, imaging centre, and dMRI head motion level as covariates.

## 3. Results

### 3.1. Hyperparameter and Model Selection

**Figure 2:**
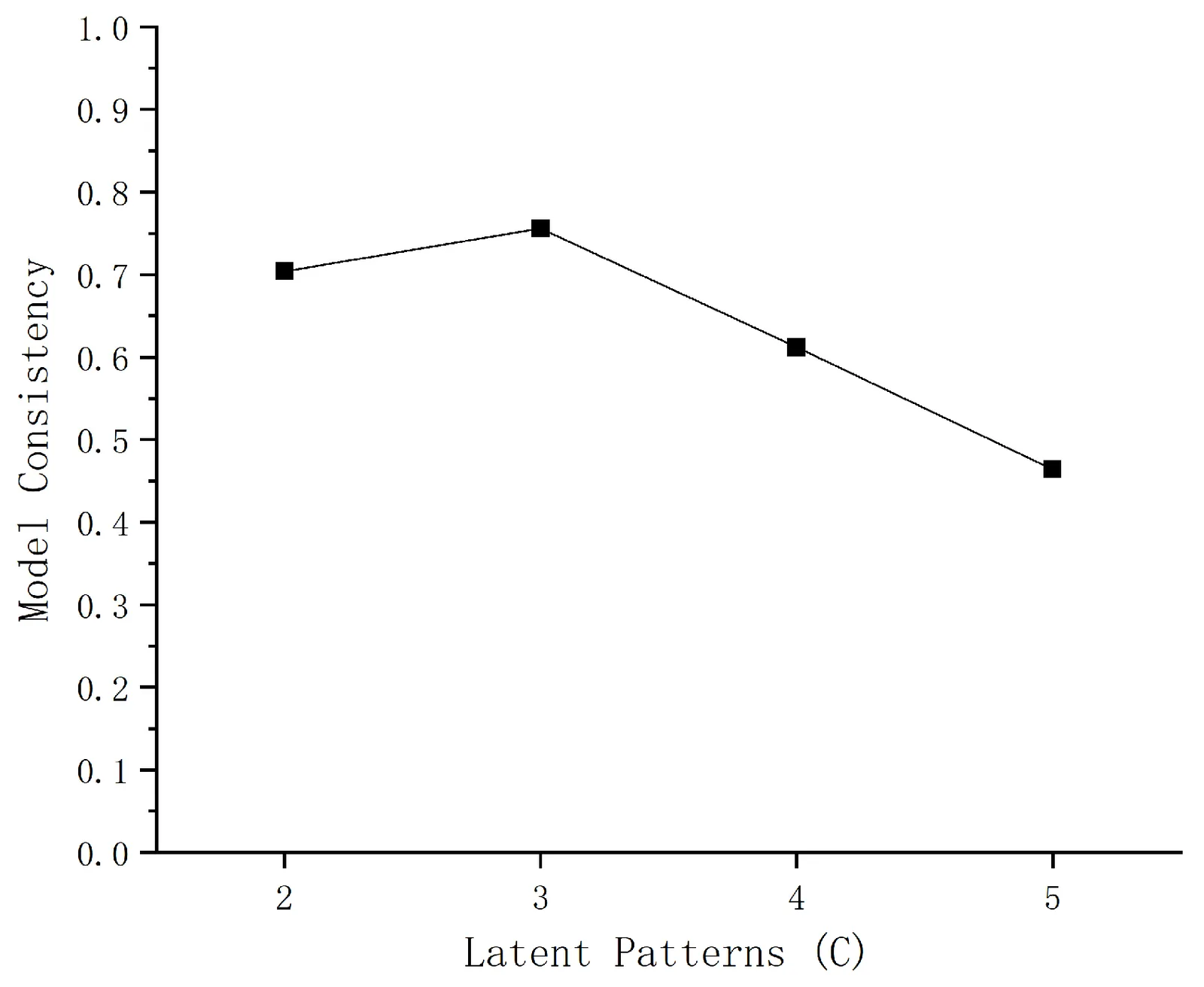
Sensitivity analysis of latent pattern (*C*).

We first evaluated the reproducibility of solutions with different numbers of patterns (C). With all other hyperparameters held at their default values, we systematically evaluated C ∈ [2, 5]. For each value of C, the model was trained 20 times, and R_indices_corr_ was calculated across these 20 runs to assess model reproducibility. The results showed that as C increased, R_indices_corr_ initially increased and then decreased, peaking at C = 3 and declining monotonically thereafter; thus, C = 3 was identified as the solution achieving the highest reproducibility under this criterion (Figure 2). With C fixed at 3, hyperparameters λ and γ were tuned via grid search, with each combination likewise trained 20 times. The highest R_indices_corr_ was obtained at λ = 0.4 and γ = 2; accordingly, the final hyperparameter configuration was set to C = 3, λ = 0.4, and γ = 2. Under this configuration, the model was independently trained 40 times to evaluate pattern stability and the robustness of the R-indices. The R_indices_corr_ across the 40 runs was 0.73, and the retained final model was trained with random seed = 16 using the checkpoint at epoch 12,000 (epoch = 12,000). It should be noted that the value reported in Table 1 corresponds to the R_indices_corr_ from the grid-search phase (20 runs), whereas 0.73 is the R_indices_corr_ across the final 40 training runs. These values are derived from different training phases, and their difference reflects normal run-to-run variability inherent in GAN retraining and should not be directly compared. Finally, the trained model was applied to the combined application cohort to characterize the WM microstructural anatomical features of each aging pattern and to examine their associations with clinical factors, lifestyle, and multidimensional phenotypic measures.

### 3.2. Statistical Validation and Collinearity Diagnostics of WM Aging Patterns

The final model identified three WM microstructural aging-related patterns, which were subsequently evaluated in the reserved set. Nested model comparisons showed that incorporating these patterns significantly improved model fit across all 144 WM metrics—the mean ΔR^2^ was 0.1897 (range: 0.0020–0.5450), 140 of 144 metrics (97.22%) showed significant improvement based on Bonferroni-corrected likelihood-ratio tests (*p* < 0.01), and AIC values decreased for every WM metric. These results should be interpreted as methodological validation rather than independent evidence, because the R-indices were derived from the same WM microstructural metrics used in the nested comparisons. To assess whether these patterns could be independently and stably included in the same multivariate model, we first examined the pairwise correlations among the three R-indices. The correlations were moderate (R_1_–R_2_: r = 0.647; R_1_–R_3_: r = 0.459; R_2_–R_3_: r = 0.575), indicating partial overlap. This partial overlap is consistent with the design of Surreal-GAN, which models coexisting continuous dimensions rather than mutually exclusive subtypes. We then computed variance inflation factors (VIFs). All VIF values fell below the conventional threshold (VIF < 5; R_1_ = 1.753, R_2_ = 2.069, R_3_ = 1.525), indicating no severe collinearity among the patterns and supporting their simultaneous inclusion in multivariate regression models.

### 3.3. WM Microstructural Features of Aging Patterns in the Reserved Set

Based on the validation results described above (Section 3.2), we further examined the associations between the three aging patterns and each WM metric in the reserved set to comprehensively map the WM microstructural anatomical features of each pattern. All statistical tests were corrected for multiple comparisons using the Bonferroni method. The direction and number of significant associations are summarized in Table 2.

The three WM aging patterns exhibited distinct WM microstructural anatomical features across the three diffusion metrics. R_1_ was characterized by a pronounced anterior-vulnerable, posterior-preserved gradient. In anterior deep and frontal projection fibers (e.g., anterior limb of the internal capsule, superior fronto-occipital fasciculus), FA showed negative associations, whereas MD and ISOVF showed positive ones; conversely, posterior commissural and association fibers (e.g., splenium of the corpus callosum, posterior thalamic radiation, and sagittal stratum) displayed the opposite pattern (positive FA, with negative MD and ISOVF). R_2_ was characterized by a widespread, general aging load pattern. For FA, all WM tracts showing significant associations were negatively correlated; for MD and ISOVF, the vast majority of tracts exhibited positive associations, with negative associations restricted to a small subset of brainstem and primary motor pathways. R_3_ was characterized by a dissociation between limbic–frontal system and sensorimotor pathway associations. The fornix and frontal projection fibers (cingulum bundle, anterior corona radiata, superior fronto-occipital fasciculus) all showed negative FA associations. Among them, the fornix showed positive MD and ISOVF associations, whereas the superior fronto-occipital fasciculus showed negative MD and ISOVF associations; the cingulum bundle and anterior corona radiata showed negative ISOVF associations, with negative MD trends that did not reach significance. In contrast, sensorimotor-related pathways (posterior limb of the internal capsule, posterior corona radiata, superior longitudinal fasciculus) showed positive FA associations, with mixed MD and ISOVF results.

**Figure 3:**
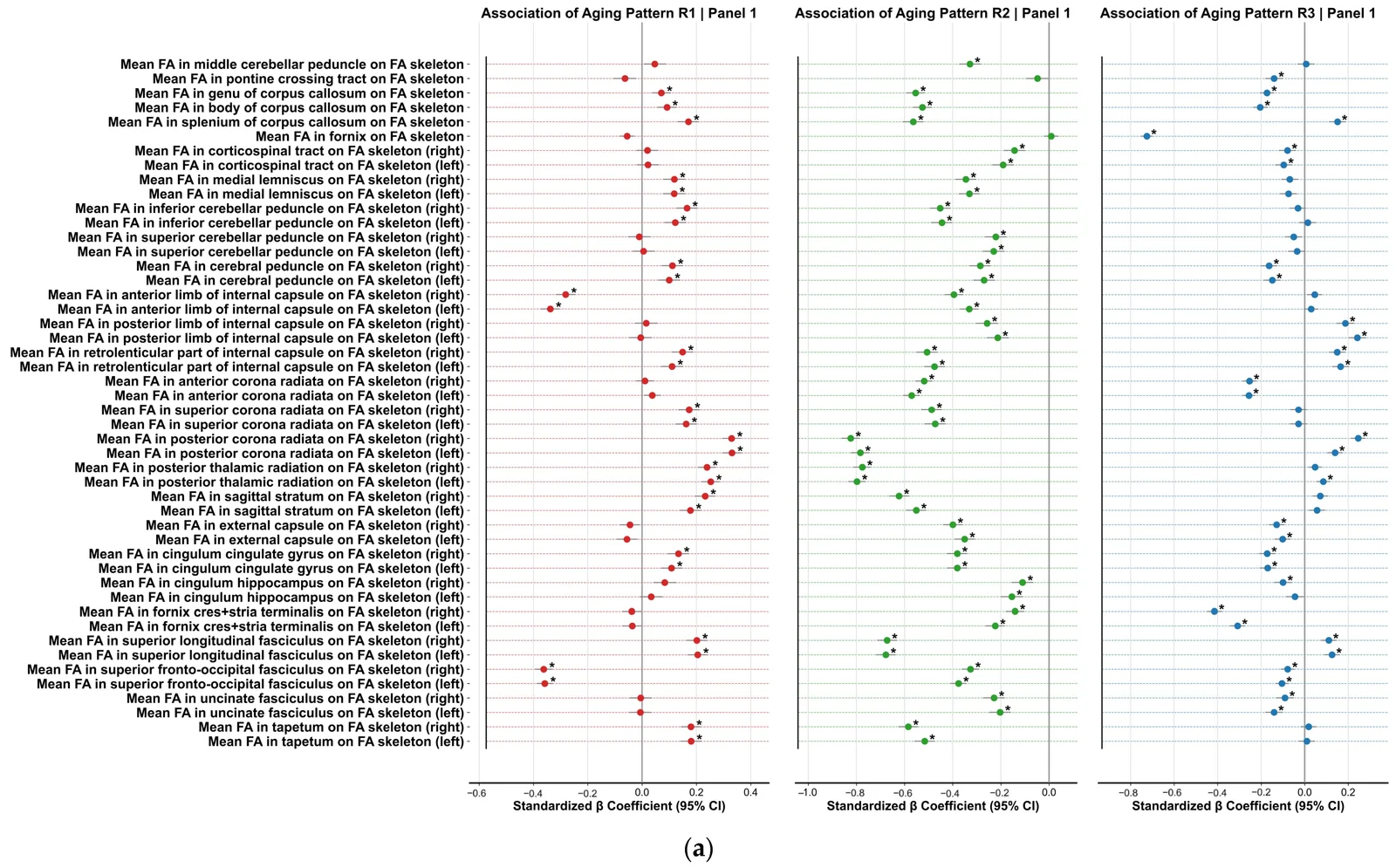

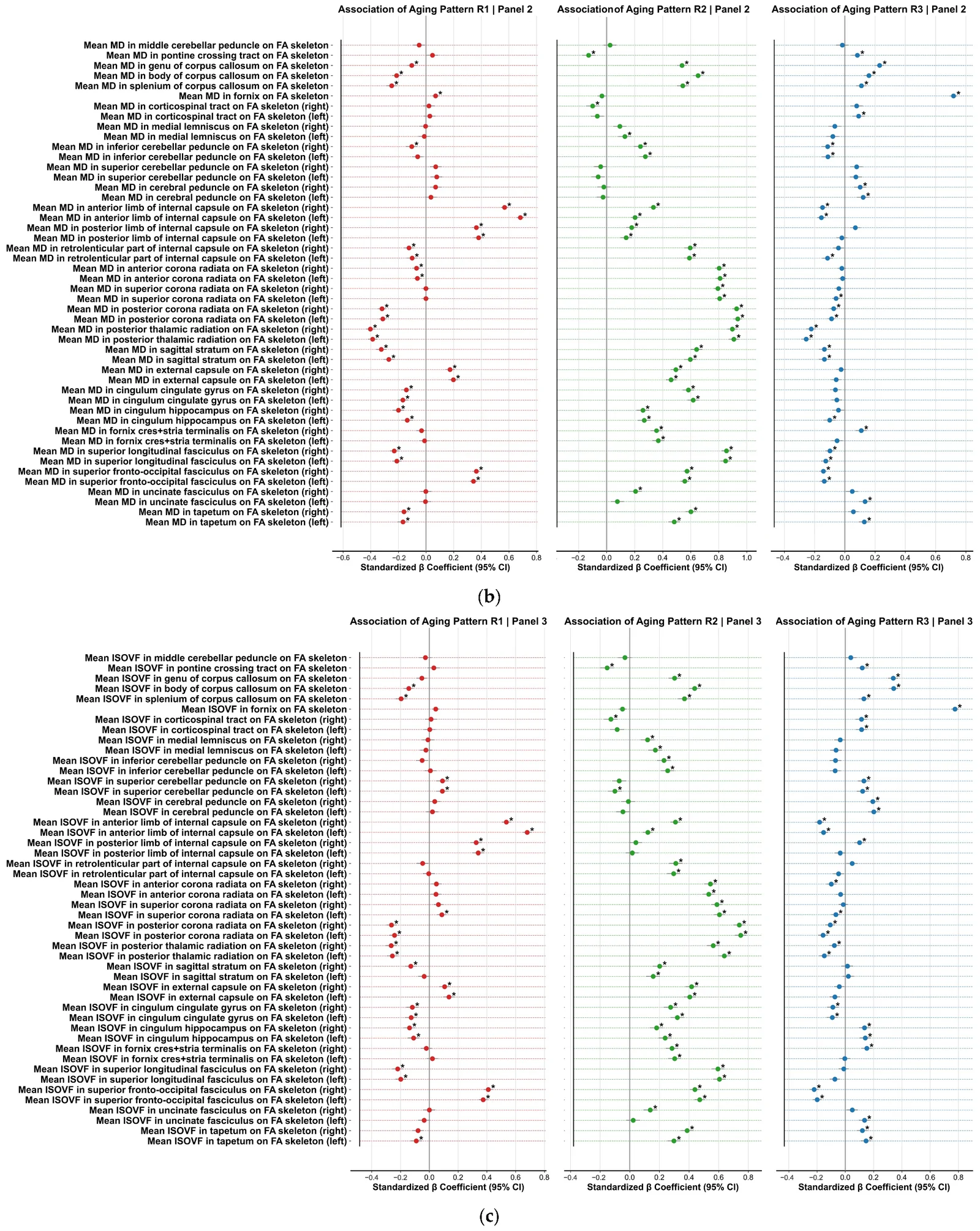
Association of WM aging patterns with WM microstructural metrics. This figure displays the statistical associations between the three WM aging patterns and the three diffusion metrics (FA, MD, and ISOVF). (**a**) 48 FA metrics, (**b**) 48 MD metrics, (**c**) 48 ISOVF metrics. R_1_ is shown in red, R_2_ in green, and R_3_ in blue. Each point represents the standardized β coefficient for a specific aging pattern in a specific WM region, with error bars indicating the corresponding 95% confidence intervals. Asterisks denote regions that remained statistically significant after correction for multiple comparisons.

Sensitivity analyses with additional adjustment for imaging centre and dMRI head motion showed that the direction of associations between the three aging patterns and WM microstructural metrics was consistent with the main analysis, and the main associations remained significant after Bonferroni correction. These results indicate that the three patterns are robust to imaging centre and head motion effects. Detailed results are provided in Supplementary Table S2. Figure 3 shows the WM microstructural features of the three patterns.

### 3.4. Symmetry Analysis of Hemispheric Aging Effects

To evaluate whether the learned aging-related representations were associated with systematic bilateral structural variation rather than being dominated by hemisphere-specific effects, we examined the topographic symmetry of R-index-associated effects between the left and right cerebral hemispheres. Standardized β coefficients for the three diffusion metrics were analyzed using correlation analyses, which assessed whether the spatial pattern of effects was consistent across hemispheres.

Correlation analyses showed that all three diffusion metrics were highly correlated between the left and right hemispheres across the three patterns, indicating strong pattern-level consistency. These results suggest that the WM aging patterns are characterized by strong bilateral symmetry, with no robust hemisphere-specific effects. Detailed statistical results are summarized in Table 3.

### 3.5. Latent Pattern Stability Analysis

To evaluate the reproducibility and stability of the WM aging patterns, cross-sample and cross-sex reproducibility analyses were conducted. Pearson correlation analyses were performed in IBM SPSS Statistics (version 27.0.1.0). Cross-sample reproducibility verification showed that all three patterns were highly correlated with their corresponding patterns in the original solution after independent retraining, indicating that the identified patterns were reproducible across variations in the training sample. Cross-sex reproducibility was assessed both within and across sexes. Within-sex validation showed high correlations for both sexes. Cross-sex cross-application (male-trained model applied to female samples and female-trained model applied to male samples) also yielded high correlations, supporting the reproducibility of the identified aging patterns across sexes. Detailed statistical results are provided in Table 4.

### 3.6. Analysis of Associated Factors in the Combined Application Cohort

Prior to the main analyses, we first examined the associations of sex, age, and educational attainment with the three aging patterns. The results showed that all three patterns were significantly associated with age. R_1_ additionally showed a significant association with educational attainment, while R_2_ and R_3_ additionally showed significant associations with sex (Table 5). Accordingly, these three factors were included as covariates in all subsequent phenotypic association analyses.

In the cognitive domain, all three aging patterns were significantly associated with slower processing speed. In addition, R_2_ and R_3_ exhibited a broader impairment profile, with their associations extending to executive function and memory. In the lifestyle domain, all three patterns showed significant associations with smoking and alcohol intake, with stronger associations observed for R_2_ and R_3_; physical activity followed a similar trend. Furthermore, R_1_ was additionally associated with diet and sleep, R_2_ with sexual factors and sleep, and R_3_ with diet and sexual factors. For physical measures, all three patterns were significantly associated with cardiovascular health, respiratory function, and physical fitness. Among them, R_2_ showed the strongest and most widespread associations with cardiovascular health, followed by R_1_; R_1_ exhibited the most prominent associations with respiratory function, followed by R_2_; and all three patterns showed comparable associations with physical fitness. In the blood biomarker domain, all three patterns were associated with alterations in glucose metabolism, liver function, and renal function: the effects on glucose metabolism were strongest for R_2_, whereas the effects on renal function were most pronounced for R_3_. R_1_ and R_2_ additionally showed significant associations with lipid metabolism markers, while R_2_ and R_3_ were jointly associated with inflammatory markers. Notably, R_3_ further exhibited a profile of hormonal metabolic abnormalities.

For blood cell counts, all three patterns showed broad associations with hematological markers, including elevations in neutrophil, leukocyte, and reticulocyte series markers, increased monocyte and eosinophil counts, and decreased lymphocyte percentage. Additionally, R_1_ and R_2_ were also significantly associated with mental health. Detailed results are presented in Table 6 and Table 7. The phenotypic associations reported above are interpreted in the Discussion, where the profiles of R_1_, R_2_, and R_3_ are discussed in relation to their spatial and biological characteristics.

Given the correlations among the three R-indices, we further performed joint models including all three R-indices simultaneously, with additional adjustment for imaging centre and dMRI head motion. The results showed that many associations were attenuated after mutual adjustment, indicating shared variance across patterns. R_2_ retained the broadest independent associations, mainly in physical measurements, blood biochemistry, lifestyle, and cognition. R_3_ retained independent associations mainly in blood biochemistry, blood count, lifestyle, physical measurements, and cognition. R_1_ retained only a limited number of independent associations, mainly in physical measurements and lifestyle. The detailed results of the joint models are presented in Table 8, and Supplementary Table S4 further provides the results that were significant at the 0.05 level.

### 3.7. Disease Risk Association

**Figure 4:**
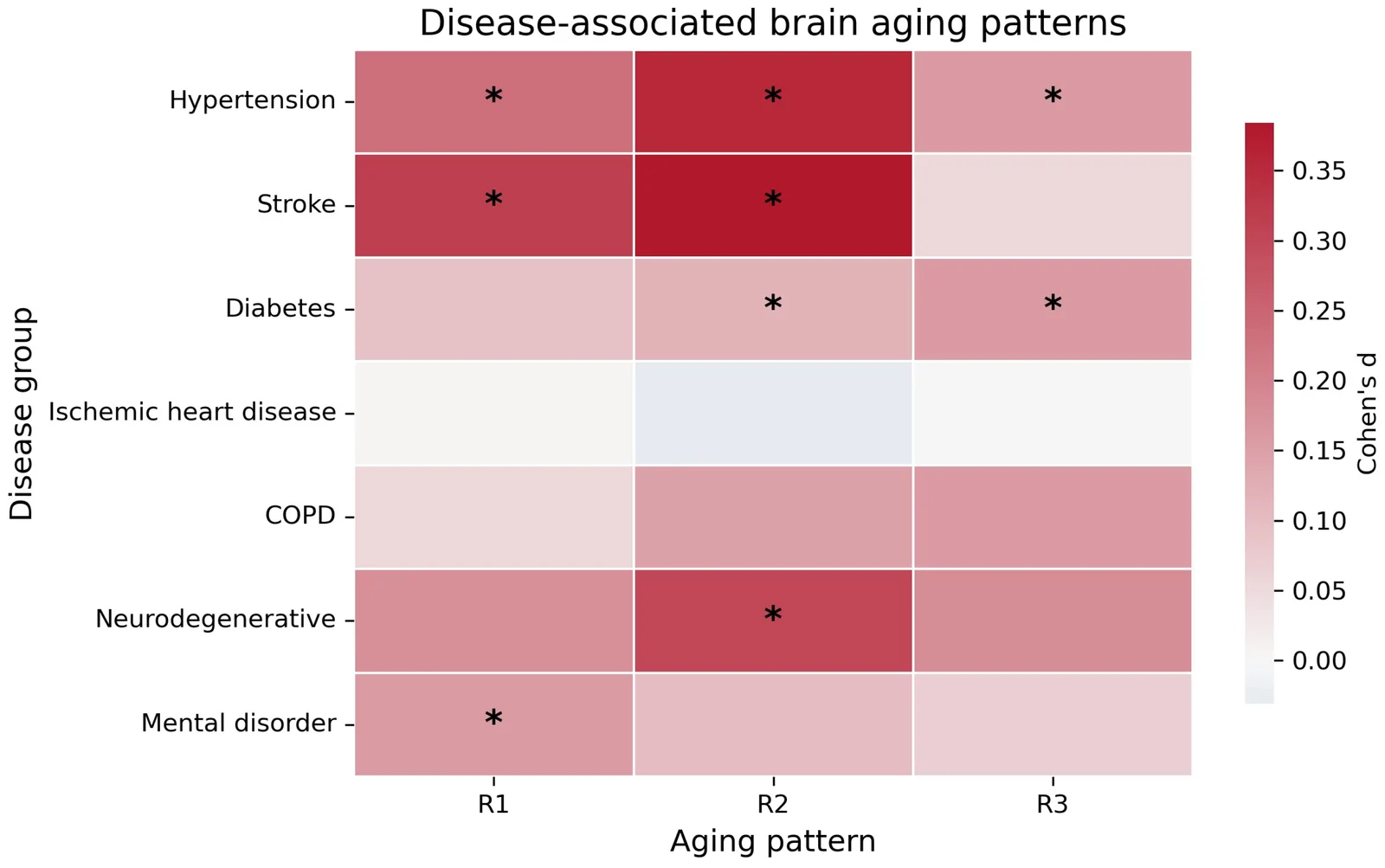
Association profiles of WM aging patterns with disease categories. “*” denotes statistically significant associations after Bonferroni correction (*p* < 0.01).

The three WM aging patterns exhibited modest but significant association profiles across the seven disease categories (Figure 4). Hypertension was the only disease significantly associated with all three patterns (R_1_: d = 0.233; R_2_: d = 0.354; R_3_: d = 0.158), with R_2_ showing the strongest association, followed by R_1_, and R_3_ exhibiting the weakest. R_1_ additionally showed significant associations with stroke (d = 0.312) and overall mental disorders (d = 0.158). R_2_ was further significantly associated with stroke (d = 0.384), diabetes (d = 0.116), and overall neurodegenerative disease (d = 0.303). R_3_ likewise demonstrated a significant association with diabetes (d = 0.156). In sensitivity analyses, after further adjusting for BMI, educational attainment, smoking status, imaging centre, and dMRI head motion in addition to sex and age, all significant associations remained robust, except for the association between R_2_ and diabetes, which was no longer significant. Given the exploratory nature of disease-category analyses and the attenuation of some associations after additional covariate adjustment, these findings should be interpreted cautiously. The disease-category associations described above are further interpreted in the Discussion. Detailed sensitivity analysis results are provided in Supplementary Table S3.

## 4. Discussion

By constructing the CDAR cohort, we applied the Surreal-GAN framework to WM microstructural imaging features to systematically characterize the latent organizational patterns of WM microstructure in clinically healthy aging. Unlike previous GAN-based studies, which have focused almost exclusively on GM atrophy or disease-related heterogeneity, the latent dimensional structure of WM microstructural aging has, to our knowledge, not yet been characterized by similar generative frameworks. The CDAR strategy substantially reduced the confounding effects of overt pathology on WM microstructure. This enabled the model to identify three coexisting but dissociable WM microstructural aging dimensions based on relatively pure aging signals. These dimensions reveal a heterogeneous landscape in which spatial topology, systemic burden, and biological processes are intertwined. Given the correlations among the R-indices, joint models showed that many phenotypic associations were shared across the three patterns; the following discussion therefore describes their phenotypic profiles without implying pattern-specificity.

### 4.1. R_1_: Differential Aging Pattern Along the Anterior–Posterior Gradient

Among the three WM aging patterns, R_1_ was characterized by a pronounced anterior–posterior gradient. Anterior WM, particularly frontal projection and association fibers, showed greater microstructural vulnerability. In contrast, posterior pathways, including the splenium of the corpus callosum and the optic radiation, were relatively preserved. This spatial organization suggests that WM aging is not uniformly distributed across the brain, but may preferentially affect fiber systems with different developmental and structural characteristics. The greater involvement of anterior association and projection pathways is consistent with the “last-in-first-out” (LIFO) framework [31,32], which proposes that neural systems that mature later in development may be particularly susceptible to age-related deterioration. Although the present cross-sectional design cannot establish that WM aging represents a reversal of developmental maturation, the spatial configuration of R_1_ is consistent with this developmental timing framework and suggests that maturational history may contribute to regional differences in WM vulnerability.

The anterior–posterior organization of R_1_ also shows conceptual convergence with the posterior–anterior shift in aging (PASA) model [33], which describes systematic age-related reorganization along the anterior–posterior axis. PASA was originally formulated from functional neuroimaging observations, and therefore does not directly explain the structural microstructural changes captured by R_1_. Nevertheless, the presence of an age-related anterior–posterior organization in WM microstructure suggests that this anatomical axis may represent a broader organizing principle of brain aging that extends across functional and structural levels. Rather than indicating that R_1_ represents a structural form of PASA, this convergence places the R_1_ pattern within a broader anterior–posterior framework of age-related brain reorganization. In this context, R_1_ extends the relevance of the anterior–posterior axis to the latent organization of WM microstructural aging. Several biological mechanisms may contribute to the preferential vulnerability of the anterior WM captured by R_1_. One possibility relates to the prolonged developmental maturation of frontal association and projection fibers. These fiber systems are among the later-maturing components of the human nervous system, with myelination continuing over an extended developmental period [34,35]. Prolonged maturation may reflect an extended period of myelin development and structural maintenance, which could contribute to later-life vulnerability under cumulative metabolic and oxidative stress. Oligodendrocytes and myelin-maintaining systems are particularly sensitive to metabolic disturbances and oxidative injury [36]. This mechanism provides a plausible biological link between developmental timing and the selective vulnerability expressed by R_1_, although it remains a mechanistic interpretation rather than a process directly demonstrated by the present data. Importantly, R_1_ was also associated with glucose metabolism measures in the present study. The convergence of a developmentally patterned spatial vulnerability with systemic metabolic associations raises the possibility that intrinsic maturational susceptibility may be modulated by cumulative metabolic burden. Given the cross-sectional design, however, the directionality and causal contribution of these metabolic pathways cannot be determined.

Beyond developmental vulnerability, systemic vascular burden may further shape the expression of R_1_. Deep and periventricular WM is supplied by relatively distal arterial territories and may therefore be particularly vulnerable to reductions in perfusion and small-vessel pathology [37]. Although this vascular anatomy does not by itself explain the entire anterior distribution of R_1_, it provides a plausible context in which systemic vascular burden could further influence the susceptibility of vulnerable WM systems. This may be relevant to R_1_ because its expression was associated with vascular risk and cerebrovascular disease, including hypertension and stroke. Rather than implying that vascular injury directly generates the R_1_ pattern, these findings suggest that systemic vascular burden may further exacerbate microstructural alterations in WM regions that are already intrinsically vulnerable because of their developmental and anatomical characteristics. Thus, the R_1_ dimension may reflect the convergence of intrinsic developmental vulnerability with extrinsic metabolic and vascular burden, rather than a single pathological mechanism. The frontal projection and association pathways implicated in R_1_ contribute substantially to processing speed and executive control, and R_1_ showed corresponding associations with these cognitive domains in the primary analysis. These cognitive associations were relatively limited and did not extend uniformly across other domains, such as memory. This profile is consistent with the anatomical organization of the affected WM systems and suggests that variation along R_1_ may be relevant to cognitive processes supported by frontal network connectivity. The correspondence between the spatial distribution of R_1_ and its cognitive correlates provides supporting evidence for the phenotypic relevance of this latent dimension.

### 4.2. R_2_: Global Microstructural Burden

In contrast to R_1_, which follows an anterior–posterior developmental gradient, R_2_ manifested as widespread microstructural aging across fiber systems. In nearly all WM metrics showing significant associations, R_2_ exhibited significant negative correlations with FA and significant positive correlations with MD and ISOVF, without being confined to specific fiber tracts or lobes. This diffuse pattern has been widely reported in dMRI studies of aging. Earlier studies have documented FA reductions in multiple WM regions in older adults, accompanied by varying combinations of radial, axial, and mean diffusivity changes. Bennett et al. [38] observed that, alongside FA reductions in older adults, there were distinct patterns of radial and axial diffusivity changes that varied across brain regions. The same group subsequently summarized evidence from DTI studies demonstrating that whole-brain FA reductions and diffusivity increases serve as robust imaging markers of WM aging [39]. Together, these studies suggest that whole-brain FA decline and diffusivity elevation are among the most robust WM imaging changes in the aging brain. Cox et al. [40] subsequently provided systematic confirmation of this global pattern in 3513 UKB participants, showing that the variance explained by a general factor across fiber tracts for FA, MD, and ISOVF increased significantly with age, indicating that the microstructural quality of different WM tracts tends to degenerate in a synchronized manner during aging. R_2_ captures this common, globally distributed aging signal as an individually expressed latent dimension. The co-occurrence of reduced FA, elevated MD, and elevated ISOVF provides a convergent microstructural signature of widespread WM deterioration. FA decline reflects reduced directional coherence of water diffusion, which can arise from changes in axonal organization, myelin integrity, or fiber geometry [41]; MD elevation is generally associated with reduced restrictions to water diffusion, while increased ISOVF indicates a greater contribution of isotropic water compartments, such as the expansion of free water spaces following tissue loosening [42]. The consistent directionality of these three metrics suggests that R_2_ reflects coordinated alterations across multiple aspects of WM microstructure rather than a change in a single diffusion property. Although these measures cannot be mapped directly onto specific histopathological processes, their combined pattern is compatible with changes involving axonal organization, myelin integrity, and tissue water distribution. This interpretation is consistent with the broader cognitive deficits associated with R_2_. Whereas R_1_ was associated primarily with processing speed and executive functions in the primary analysis, the cognitive correlates of R_2_ extended to memory domains, aligning with the disconnection framework in which diffuse WM damage impairs conduction efficiency across large-scale brain networks.

A noteworthy exception within R_2_ is that a small number of fiber tracts, mainly concentrated in the brainstem and some primary motor pathway-related regions, exhibited a pattern of reduced FA accompanied by concurrent reductions in MD and ISOVF. This deviation from the dominant whole-brain trend may arise from the superimposition of different aging-related microstructural events. One possible explanation is that region-specific tissue properties may modify the expression of age-related diffusion changes. For example, experimental evidence suggests that iron deposition can influence diffusion parameter estimates by altering relaxation and diffusion-weighted signal characteristics [43]. At the same time, age-related changes in axonal or myelin integrity could contribute to FA reductions. Thus, the divergent direction of MD and ISOVF in these regions should not necessarily be interpreted as evidence of relative preservation of microstructural integrity compared with other WM regions, but may reflect the combined influence of different microstructural and measurement-related factors. In addition to iron accumulation and gliosis, other factors such as pre-existing developmental gradients and NODDI model assumptions may also contribute to this regional divergence, although the present data do not allow these mechanisms to be distinguished directly. This also illustrates that the global burden represented by R_2_ is not a homogeneous amplification of a single pathological process, but rather the result of multiple microstructural alterations superimposed in different ways across regions.

R_2_ exhibited broader disease associations, being significantly associated with hypertension, stroke, diabetes, and overall neurodegenerative disease burden. This pattern is consistent with its peripheral phenotypic profile: R_2_ showed widespread and robust associations with metabolic, inflammatory, and vascular markers, suggesting that it represents the cumulative projection of multi-system risks—including cardiovascular, metabolic, and inflammatory factors—onto WM. In this way, R_2_ transforms what has previously been described as “whole-brain WM aging” at the group-average level into an individually quantifiable continuous burden dimension. It also reveals that, even against a background of global aging, local microstructural components can undergo remodeling in divergent directions.

### 4.3. R_3_: Dissociated Aging Patterns Between the Limbic–Frontal and Sensorimotor Systems

The hallmark of R_3_ was a dissociated pattern of microstructural changes between and within the limbic–frontal and sensorimotor systems. This inconsistency in the combination of diffusion metrics across and within systems suggests that R_3_ captures the differential aging expression of distinct functional systems at the microstructural level.

The limbic–frontal system is a highly distributed network whose different pathways serve distinct functional modules, including episodic memory, executive control, and emotion regulation. Aging may not act uniformly across all pathways, but may preferentially affect connections that serve as information convergence or hub functions within the network [44] and has been identified as a WM pathway that is particularly susceptible to age-related microstructural alterations [45]. In contrast, the superior fronto-occipital fasciculus and the anterior corona radiata, although also belonging to the frontal system, have more distributed connection targets and may respond to aging in different ways, manifesting as complex combinations of diffusion metrics. This intra-system heterogeneity indicates that aging of the limbic–frontal system is not a unitary degenerative process, but may instead reflect differences in the structural organization and connectivity of its constituent pathways.

In the sensorimotor system, several pathways, such as the posterior corona radiata, exhibited a pattern of elevated FA with MD/ISOVF that did not increase but instead decreased; this pattern cannot be simply interpreted as a structural benefit. Rather, changes in FA may reflect alterations in local fiber architecture, including changes in fiber orientation and the relative contribution of crossing fibers, rather than preservation of tissue integrity per se [46]. Similarly, the absence of an increase—or even a decrease—in MD/ISOVF should not be directly equated with tissue health; it may instead reflect alterations in the local water diffusion environment or tissue composition, which do not correspond simply to functional status. Thus, the atypical diffusion signature observed in these pathways should be interpreted as a distinct microstructural expression of aging rather than evidence of structural preservation. At the same time, proximal motor pathways such as the cerebral peduncle still displayed the conventional degenerative direction of reduced FA accompanied by elevated MD/ISOVF, contrasting with the atypical pattern described above. This intra-system divergence within the sensorimotor system suggests that its aging does not simply proceed along a developmental gradient, but is instead shaped more by differences in fiber architecture, connectivity, and regional tissue properties.

The associations of R_3_ with peripheral phenotypes related to metabolism, hormones, and renal function suggest that this system-level dimension may reflect the differential relationship between systemic biological factors and microstructural variation across functional systems. This system-level organization indicates that the heterogeneity of WM aging is also manifested in the differential responses of different functional systems to common aging pressures. In this respect, R_3_ adds a system-level dimension to WM aging heterogeneity: whereas R_1_ emphasizes regional vulnerability along an anterior–posterior axis and R_2_ captures a globally distributed aging burden, R_3_ highlights the dissociation of aging-related microstructural changes across functional systems and their constituent pathways. R_3_ thus provides a framework for considering WM aging as a process that is organized not only spatially, but also according to the structural and functional architecture of distributed brain systems. However, this dimension currently lacks direct molecular or histological evidence. Therefore, the system-level aging suggested by R_3_ should be regarded as a testable interpretive framework rather than an established biological conclusion.

### 4.4. Limitations and Future Directions

This study has several inherent limitations. First, all participants were drawn from the UKB database, which predominantly comprises middle-aged and older volunteers of white British ethnicity who were community-dwelling and not recruited from clinical or institutional settings, with relatively high socioeconomic status and health awareness. The imaging subsample may also be subject to healthy-volunteer bias. Caution is therefore warranted when generalizing this analytical framework to other populations; future studies should validate its generalizability in more diverse, multi-center cohorts. Second, owing to the cross-sectional design, the identified R-indices reflect between-group differences across age strata rather than within-individual longitudinal aging trajectories. Future integration of longitudinal data will help to further verify the trajectory stability of these dimensions.

At the methodological level, although the CDAR cohort was rigorously screened for overt clinical pathology based on ICD-10 codes, potential influences such as clinically silent cerebral small vessel disease, early protein deposition, or long-term medication history cannot be fully excluded. These factors may confound the three identified dimensions. Future work combining the CDAR framework with biomarkers is expected to further strengthen the biological interpretability and robustness of the three patterns, thereby rendering the derived aging dimensions more compelling. A further limitation is that the model was trained on discrete age anchors (≤55 vs. ≥65 years). The R-indices were therefore validated only in the ≥65-year application cohort, and whether they behave as meaningful continuous measures in the 55–65-year transitional range or below 55 years remains untested. Future continuous-age or longitudinal studies are needed to assess their generalizability across the adult lifespan. In addition, although both DTI and NODDI metrics were employed, all regional extraction was based on the TBSS skeleton and the JHU atlas, which have limited resolving power for fine fiber tracts and crossing-fiber regions. Future studies may benefit from finer-grained WM atlases and tract-specific analytical approaches. Finally, the phenotypic and disease association analyses can only reveal correlational relationships and cannot establish causation; future integration of causal inference methods such as Mendelian randomization represents an important extension of this work.

## 5. Conclusions

In the CDAR cohort, we used the Surreal-GAN framework to disentangle three reproducible and coexisting aging patterns from WM microstructural data: R_1_, characterized by an anterior vulnerability–posterior preservation gradient; R_2_, characterized by widespread diffuse aging burden; and R_3_, characterized by a dissociation between the limbic–frontal system and sensorimotor pathways. The three patterns were anatomically dissociable, with partially overlapping phenotypic profiles but differing independent associations. These findings indicate that WM aging comprises multiple dissociable latent processes rather than a single trajectory. Together, R_1_, R_2_, and R_3_ suggest that WM aging is organized across complementary levels of regional vulnerability, global microstructural burden, and system-level differentiation. These findings further support a dimensional framework for characterizing brain aging heterogeneity at the WM microstructural level. More broadly, this framework provides a basis for moving beyond population-average descriptions of WM aging toward individualized characterization of how aging-related microstructural variation is spatially and systemically organized, and may ultimately help define more informative imaging reference dimensions against which early pathological alterations can be evaluated.

## Figures and Tables

**Table 1:** Model consistency under different hyperparameter combinations.

	λ = 0.05	λ = 0.2	λ = 0.4	λ = 0.8
γ = 0.01	0.581	0.577	0.666	0.674
γ = 2	0.791	0.807	**0.827**	0.760
γ = 6	0.738	0.727	0.740	0.728

Notes: These results were obtained under C = 3. For each hyperparameter combination, the model was trained independently 20 times, and the corresponding R_indices_corr_ is reported as the consistency metric. The optimal result is highlighted in bold.

**Table 2:** Summary of WM aging patterns and microstructural associations.

Measure	R-Index	Significant (n/N)	Direction (neg/pos)
FA	R_1_	29/48	4 neg/25 pos
	R_2_	46/48	46 neg/0 pos
	R_3_	31/48	21 neg/10 pos
MD	R_1_	31/48	22 neg/9 pos
	R_2_	39/48	2 neg/37 pos
	R_3_	28/48	17 neg/11 pos
ISOVF	R_1_	25/48	14 neg/11 pos
	R_2_	39/48	3 neg/36 pos
	R_3_	30/48	12 neg/18 pos

Note: FA, MD, and ISOVF metrics each comprised 48 WM tract regions, yielding 144 WM microstructural metrics in total. Significance was determined after Bonferroni correction (*p* < 0.01). “pos” indicates a positive association, and “neg” indicates a negative association.

**Table 3:** Pearson correlation analysis results.

Pattern	Variable Pair	Pearson’s *r*	*p*	*p_adj* (Bonferroni)	95% CI Lower	95% CI Upper
R_1_	FA_L vs. FA_R	0.990	1.91 × 10^−17^	1.72 × 10^−16^	0.974	0.996
	MD_L vs. MD_R	0.991	3.40 × 10^−18^	3.06 × 10^−17^	0.978	0.997
	ISOVF_L vs. ISOVF_R	0.981	4.51 × 10^−15^	4.06 × 10^−14^	0.954	0.993
R_2_	FA_L vs. FA_R	0.978	2.38 × 10^−14^	2.14 × 10^−13^	0.945	0.991
	MD_L vs. MD_R	0.988	9.00 × 10^−17^	8.10 × 10^−16^	0.969	0.995
	ISOVF_L vs. ISOVF_R	0.969	5.45 × 10^−13^	4.91 × 10^−12^	0.924	0.988
R_3_	FA_L vs. FA_R	0.960	5.79 × 10^−12^	5.21 × 10^−11^	0.902	0.984
	MD_L vs. MD_R	0.868	3.49 × 10^−7^	3.14 × 10^−6^	0.697	0.945
	ISOVF_L vs. ISOVF_R	0.865	4.25 × 10^−7^	3.83 × 10^−6^	0.691	0.944

Note: *p_adj* indicates Bonferroni-corrected *p*-values. All correlations remained significant after correction.

**Table 4:** Model reproducibility results.

Validation	Subgroup	Pattern	Pearson’s *r*	*p*
Cross-sample reproducibility	—	R_1_	0.929	<0.001
		R_2_	0.887	<0.001
		R_3_	0.935	<0.001
Cross-sex reproducibility	male-trained to male	R_1_	0.937	<0.001
		R_2_	0.902	<0.001
		R_3_	0.916	<0.001
	female-trained to female	R_1_	0.929	<0.001
		R_2_	0.894	<0.001
		R_3_	0.891	<0.001
	male-trained to female	R_1_	0.925	<0.001
		R_2_	0.894	<0.001
		R_3_	0.930	<0.001
	female-trained to male	R_1_	0.904	<0.001
		R_2_	0.899	<0.001
		R_3_	0.903	<0.001

**Table 5:** Associations of demographic covariates with the three WM aging patterns.

	R_1_	R_2_	R_3_
Age	<0.001 *	<0.001 *	<0.001 *
Sex	0.98	<0.001 *	<0.001 *
Education	0.017 *	0.73	0.11

“*” indicates statistically significant results.

**Table 6:** Associations between cognitive function and aging patterns.

Cognitive Domain	Cognitive Test	Variable	Type	Subjects	R_1_	R_2_	R_3_
Visual declarative Memory	Pairs Matching Test	Time to complete round (Round 1)	continuous ^1^	11,169	— ^2^	—	0.05
		Time to complete round (Round 2)	continuous	11,187	—	—	0.04
Prospective memory	Prospective Memory Test	Correct recall on first attempt	binary ^3^	11,476	—	0.67	0.59
		Number of attempts	count ^4^	11,362	—	1.16	1.15
Verbal declarative Memory	Paired Associate Learning	Number of word pairs correctly associated	count	8668	—	0.93	—
Executive function	Tower Rearranging Test	Number of puzzles correct	count	8386	—	—	0.94
		Number of puzzles attempted	count	8668	—	0.95	—
	Trail Making Test (Part B)	Total errors traversing alphanumeric path	count	8340	1.12	1.14	1.19
		Duration to complete alphanumeric path	continuous	8129	—	0.07	0.06
	Trail Making Test (Part A)	Duration to complete numeric path	continuous	8509	—	0.06	0.06
Processing speed	Symbol Digit Substitution Test	Number of symbol digit matches made correctly	count	8515	0.94	0.9	0.91
		Number of symbol digit matches attempted	count	8515	0.95	0.91	0.91
	Reaction Time Test	Mean time to correctly identify matches	continuous	11,360	0.05	0.07	0.07

Note: All reported values reached statistical significance after Bonferroni correction (*p* < 0.01). Effect sizes are reported according to variable type: ^1^ Continuous variables: effect sizes are expressed as standardized β coefficients, with values > 0 indicating a positive association and values < 0 indicating a negative association; ^2^ “—” denotes non-significance after correction (*p* ≥ 0.01); non-significant variables are not listed in the table; ^3^ Binary variables: effect sizes are expressed as odds ratios (OR), with values > 1 indicating a positive association and values < 1 indicating a negative association; ^4^ Count variables; effect sizes are reported as IRR, with values greater than 1 indicating positive associations and values less than 1 indicating negative associations.

**Table 7:** Associations between phenotypes and aging patterns.

Dimension	Component	Variable	Type	Subjects	R_1_	R_2_	R_3_
Lifestyle	Diet	Cereal intake	continuous	13,737	—	—	−0.045
		Processed meat intake	ordinal ^5^	13,742	1.35	—	—
		Bread intake	continuous	13,679	—	—	−0.041
	Smoking	Pack years of smoking	continuous	3870	0.072	0.108	0.091
		Age stopped smoking	continuous	3903	0.077	0.124	0.108
		Ever smoked	binary	13,671	—	—	1.35
	Alcohol intake	Mean weekly alcohol intake	continuous	9666	0.051	0.091	0.12
	Sleep	Getting up in morning	ordinal	13,745	0.76	—	—
		Nap during day	ordinal	13,751	—	1.34	—
	Physical activity	Usual walking pace	ordinal	13,721	0.6	0.47	0.48
	Sexual factors	Lifetime number of sexual partners	count	10,880	—	1.75	1.55
Physical measurements	Cardiovascular risk factors	Diastolic blood pressure	continuous	10,843	0.123	0.146	0.071
		Systolic blood pressure	continuous	10,834	0.108	0.144	0.091
		Pulse rate	continuous	9658	0.074	0.059	—
		Waist circumference	continuous	13,467	0.09	0.115	0.086
		Body mass index (BMI)	continuous	13,423	0.09	0.128	0.089
		Hip circumference	continuous	13,469	0.052	0.072	—
		Maximum carotid IMT (intima-medial thickness)	continuous	11,812	0.042	0.062–0.075	—
		Mean carotid IMT	continuous	11,812	—	0.053–0.069	—
		Pulse wave reflection index	continuous	11,551	—	−0.045	−0.064
	Respiratory function	Forced vital capacity (FVC)	continuous	7999	−0.077	−0.061	−0.054
		Forced expiratory volume in 1 s (FEV1)	continuous	7999	−0.066	−0.056	−0.053
	Physical fitness	Hand grip strength (left)	continuous	13,408	−0.069	−0.068	−0.07
		Hand grip strength (right)	continuous	13,403	−0.079	−0.072	−0.075
Blood biochemistry	Glucose metabolism	Glycated haemoglobin (HbA1c)	continuous	12,914	0.05	0.063	0.055
		Glucose	continuous	11,854	0.053	0.062	0.055
	Liver function	Gamma glutamyltransferase	continuous	12,957	—	0.045	0.044
		Alanine aminotransferase	continuous	12,968	0.044	0.049	—
	blood lipid	Triglycerides	continuous	12,963	0.047	—	—
		Cholesterol	continuous	12,972	—	−0.045	—
		low-density lipoprotein (LDL) direct	continuous	12,937	—	−0.045	—
	Renal function	Urate	continuous	12,960	0.039	0.049	0.073
	Inflammatory markers	C-reactive protein	continuous	12,943	—	0.048	0.048
	hormone	insulin-like growth factor 1 (IGF-1)	continuous	12,913	—	—	−0.049
Blood count	White blood cell series	Neutrophill count	continuous	13,251	0.06	0.073	0.077
		White blood cell (leukocyte) count	continuous	13,244	0.05	0.065	0.07
		Monocyte count	continuous	13,215	0.059	0.056	0.054
		Eosinophill count	continuous	13,215	—	0.052	0.044
		Lymphocyte percentage	continuous	13,215	−0.039	−0.043	—
	Reticulocytes/Erythrocytes	High light scatter reticulocyte percentage	continuous	13,048	0.073	0.069	0.09
		High light scatter reticulocyte count	continuous	13,048	0.071	0.067	0.088
		Immature reticulocyte fraction	continuous	13,048	0.058	0.055	0.063
		Reticulocyte percentage	continuous	13,048	0.058	0.046	0.066
		Reticulocyte count	continuous	13,048	0.056	0.044	0.063
Psychosocial factors	Mental health	Neuroticism score	continuous	11,573	0.045	—	—
		Mood swings	binary	13,423	—	1.47	—

^5^ Ordinal categorical variables: effect sizes are expressed as odds ratios (OR), with values > 1 indicating a positive association and values < 1 indicating a negative association. For reporting conventions of other variable types, see Table 6 notes.

**Table 8:** Phenotypic associations of the three WM aging patterns in joint models.

Dimension	Component	Variable	Type	Subjects	R_1_	R_2_	R_3_
Lifestyle	Smoking	Age stopped smoking	continuous	3903	—	0.098	—
	Alcohol intake	Mean weekly alcohol intake	continuous	9666	—	—	0.099
	Physical activity	Usual walking pace	ordinal	13,721	—	0.596	0.651
	Sexual factors	Lifetime number of sexual partners	count	10,880	0.747	1.736	1.24
Physical measurements	Cardiovascular risk factors	Systolic blood pressure	continuous	10,834	—	0.126	—
		Diastolic blood pressure	continuous	10,843	—	0.122	—
		Pulse rate	continuous	9658	0.059	—	—
		Waist circumference	continuous	13,467	—	0.093	—
		BMI	continuous	13,423	—	0.106	—
		Hip circumference	continuous	13,469	—	0.078	—
		Mean carotid IMT	continuous	11,901	—	0.075–0.083	—
		Maximum carotid IMT	continuous	11,901	—	0.068–0.086	—
		Pulse wave reflection index	continuous	11,551	—	—	−0.05
	Respiratory function	FVC	continuous	7999	−0.059	—	—
	Physical fitness	Hand grip strength (right)	continuous	13,403	−0.034	—	−0.05
		Hand grip strength (left)	continuous	13,408	—	—	−0.048
Blood biochemistry	Blood lipid	Cholesterol	continuous	12,972	—	−0.054	—
	Renal function	Urate	continuous	12,960	—	—	0.062
Blood count	White blood cell series	Neutrophil count	continuous	13,215	—	—	0.052
		White blood cell (leukocyte) count	continuous	13,244	—	—	0.048
	Erythrocytes	High light scatter reticulocyte percentage	continuous	13,048	—	—	0.067
		High light scatter reticulocyte count	continuous	13,048	—	—	0.067
		Reticulocyte percentage	continuous	13,048	—	—	0.051
		Reticulocyte count	continuous	13,048	—	—	0.049
Cognitive	Processing speed	Number of symbol digit matches made correctly	count	8515	—	0.924	0.94
		Number of symbol digit matches attempted	count	8515	—	0.933	0.945

Note: Only associations with Bonferroni-corrected *p* < 0.01 in joint models are shown. For reporting conventions of variable types, see Table 6 and Table 7 notes.

## Data Availability

The imaging data analyzed in this study were obtained from the UK Biobank and are available through the UK Biobank Access Management System (http://www.ukbiobank.ac.uk/register-apply/; accessed on 20 September 2025). Access to these data is governed by the UK Biobank Research Access Administration Team and is open to both academic and commercial applicants under the same review procedures. Applications are assessed based on their relevance to health-related research objectives. Additional information regarding available datasets can be found on the UK Biobank website (http://www.ukbiobank.ac.uk; accessed on 20 September 2025). Owing to ongoing updates to the resource, the number of participants with available imaging data may differ slightly from the sample reported in this study. The Surreal-GAN framework is publicly available from Yang et al. (https://github.com/zhijian-yang/SurrealGAN; accessed on 20 September 2025). The hyperparameter configuration used in this study is fully reported in Section 2.2.3 Model Training and Hyperparameter Optimization and Section 3.1 Model Selection. Analysis code is available from the corresponding author upon reasonable request.

## Supplementary Materials

The following supporting information can be downloaded at https://www.mdpi.com/article/10.3390/brainsci16101076/s1: Table S1: ICD-10 exclusion codes used to define the clinically screened healthy CDAR cohort; Table S2: S2a. Detailed statistical results for associations between WM microstructural metrics and the three aging patterns, S2b. Sensitivity analysis of associations between WM microstructural metrics and the three aging patterns after additional adjustment for imaging centre and dMRI head motion; Table S3: Sensitivity analyses for disease associations with additional adjustment for imaging centre and dMRI head motion; Table S4: Independent phenotypic associations of the three WM aging patterns in joint models adjusted for imaging centre, head motion, and other R-indices.

## Abbreviations

The following abbreviations are used in this manuscript:

Abbreviation	Full Name
ABA	accelerated brain aging
AIC	Akaike information criterion
AMICO	accelerated microstructure imaging via convex optimization
BMI	body mass index
CDAR	clinically defined aging reference
CDAR-REF	CDAR reference group
CDAR-TAR	CDAR target group
CI	confidence interval
CNS	central nervous system
COPD	chronic obstructive pulmonary disease
DAPH	dissociable aging–pathology hypothesis
dMRI	diffusion MRI
DTI	diffusion tensor imaging
DTIFIT	diffusion tensor fitting
Eddy	FSL eddy current and motion correction tool
EPI	echo planar imaging
FA	fractional anisotropy
FEV1	forced expiratory volume in 1 s
fMRI	functional MRI
FNIRT	FMRIB’s nonlinear image registration tool
FSL	FMRIB software library
FVC	forced vital capacity
GAN	generative adversarial network
GLM	generalized linear model
GM	gray matter
HbA1c	glycated hemoglobin
HDC	health-to-disease continuum
ICD-10	International Classification of Diseases, 10th Revision
IDP	imaging derived phenotype
IHD	ischemic heart disease
IGF-1	insulin-like growth factor 1
IRR	incidence rate ratio
ISOVF	isotropic water volume fraction
JHU	Johns Hopkins University
LDL	low-density lipoprotein
LIFO	last-in-first-out
MD	mean diffusivity
MNI152	Montreal Neurological Institute 152
MRI	magnetic resonance imaging
NODDI	neurite orientation dispersion and density imaging
non-IDP	non-imaging derived phenotype
OR	odds ratio
PASA	posterior–anterior shift in aging
RBA	resilient brain aging
REF	reference group
rfMRI	resting-state functional MRI
R-indices	latent dimensions
R_indices_corr_	R-indices correlation
SD	standard deviation
SPSS	Statistical Package for the Social Sciences
Surreal-GAN	semi-supervised representation learning via GAN
SWI	susceptibility-weighted imaging
T1w MRI	T1-weighted MRI
T2-FLAIR	T2-weighted fluid-attenuated inversion recovery
TAR	target group
TBSS	tract-based spatial statistics
tfMRI	task functional MRI
TOPUP	FSL susceptibility-induced distortion correction tool
UKB	UK Biobank
VIF	variance inflation factor
WM	white matter
WMH	white matter hyperintensity
